# Correlation of clone size with clinical and lab presentation in Paroxysmal nocturnal hemoglobinuria (PNH): A single center experience

**DOI:** 10.12669/pjms.41.9.11897

**Published:** 2025-09

**Authors:** Memoona Khan, Nabeela Khan, Saima Humayun, Haider Nisar, Mehreen Ali Khan

**Affiliations:** 1 Memoona Khan Consultant Hematologist, Armed Forces Bone Marrow Transplant Center, National Institute of Bone Marrow Transplant, CMH Medical Complex, Rawalpindi, Pakistan; 2 Nabeela Khan Consultant Hematologist, Armed Forces Bone Marrow Transplant Center, National Institute of Bone Marrow Transplant, CMH Medical Complex, Rawalpindi, Pakistan; 3 Saima Humayun Consultant Hematologist and BMT Physician, Armed Forces Bone Marrow Transplant Center, National Institute of Bone Marrow Transplant, CMH Medical Complex, Rawalpindi, Pakistan; 4 Haider Nisar Consultant Pediatrician and BMT Physician, Armed Forces Bone Marrow Transplant Center, National Institute of Bone Marrow Transplant, CMH Medical Complex, Rawalpindi, Pakistan; 5 Mehreen Ali Khan Consultant Hematologist and BMT Physician, Armed Forces Bone Marrow Transplant Center, National Institute of Bone Marrow Transplant, CMH Medical Complex, Rawalpindi, Pakistan

**Keywords:** Clone, Fluorescein labelled proaerolysin, Paroxysmal nocturnal hemoglobinuria

## Abstract

**Objective:**

To determine the correlation between clone size in Paroxysmal nocturnal hemoglobinuria and clinical presentation.

**Methodology:**

A prospective study was conducted in Armed Forces Bone Marrow Transplant Center (AFBMTC), between 2021-2024. PNH testing was done by multiparametric flow cytometry using fluorescently labeled Alexa-488. PNH clone was labelled as large (>50%) or small (<30%) based on ASH education program 2021. Clone size was correlated with type of presentation of PNH and hemolysis. Statistical analyses were performed using SPSS v 25.0.

**Results:**

Out of 324 samples, 96 (29.6 %) were found to have PNH clone by FLAER. Among 75 evaluable cases, 55 (71.2%) were diagnosed as classical hemolytic PNH(CPNH), 16 (21.3%) as PNH in context of marrow failure, 3 (4.2%) as aplastic anemia transformed into PNH, 1 (1.4%) case of MDS with PNH clone. Mean age of patients was 30.97 + 10.56 years. 60 (82.2%) patients were male while 15 (17.3%) were female. On granulocytes, large clone (>50%) was found in 81.3% cases and small clone (<30%) in 18.6% cases. Similarly, 73.3% cases had a large clone on monocytes while 26.6% had a small clone. However, most cases (62.6%) had a smaller RBC clone size. Granulocytic and monocytic clone sizes were significantly associated with Hb and platelet count (p- value <0.05). However, RBC clone size did not show any significant association with the blood counts. Similarly, no significant correlation could be found between markers of hemolysis/ thrombosis.

**Conclusion:**

Size of PNH clone impacts the clinical presentation with large PNH clones typically seen in CPNH.

## INTRODUCTION

Paroxysmal nocturnal hemoglobinuria is a rare acquired disorder of glycosylphosphatidylinositol (GPI) -linked proteins’ deficient hematopoietic stem cells. The underlying pathophysiology includes somatic mutation in X-linked phosphatidylinositol (PIG) gene, Class A. Subsequent to the mutation, there is complete or partial deficiency of GPI anchored proteins from various cell surfaces. Clinical spectrum of the disorder varies from episodes of hemolysis and failure of bone marrow to significant thrombosis.^1^-^3^

Fluorescein labelled proaerolysin (FLAER) is a highly sensitive standardized multi parametric flow cytometry assay to detect PNH clones in suspected cases.^4^ The technique is increasingly being used over the past 10 years to detect PNH clones in cases of suspected bone marrow failure syndromes. The technique has the reported sensitivity of > 0.01%. These small clone sizes can be easily missed by multiparametric flow cytometry (MP-FCM) and is also affected by recently transfused red cells and congenital deficiencies of GPI anchored proteins like CD55.^5^ The assay is based on binding of flourescein labelled proaerolysin to GPI anchor on the cell membranes. The assay has been developed in accordance with 2010 International Clinical Cytometry Society (ICCS) PNH Consensus Guidelines and 2012 Practical PNH guidelines.^6^,^7^

About 50 to 60% Aplastic anemia patients in western population have been reported to have a PNH clone^8^, the percentage reported in Indians is 41%^9^ while 60.9% patients of aplastic anemia in Pakistani population were reported to have PNH clone by our center.^5^ The significance of PNH clone in relation to the disease type, clinical presentation and response to immunosuppressive therapy (IST) has been evaluated in some studies previously.^1^,^4^ However, this study aimed to document the utility of FLAER based testing for the diagnosis of PNH and to determine the correlation between clone size and clinical presentation in Pakistani population.

## METHODOLOGY

This was a single center prospective study conducted at Armed Forces Bone Marrow Transplant Center/National Institute of Bone Marrow transplant (AFBMTC/NIBMT) between 2021 to 2024.

### Ethical Approval:

It was obtained from the hospital ethics committee (Ref: IRB/032/AFBMTC/Approval/2025; dated January 14, 2021). Informed written consent was taken from all the enrolled patients.

After detailed clinical history and examination, samples for complete hemogram, markers of hemolysis (LDH, bilirubin), bone marrow aspirate and trephine and samples for PNH testing of suspected PNH cases were obtained and sent to relative sections of laboratory department of AFBMTC. All samples submitted for analysis of PNH clone were subject to FLAER analysis in the flow cytometry department of AFBMTC. Cases, found to have a PNH clone, were diagnosed either as PNH (subclassified as Classical hemolytic PNH or PNH in context of marrow failure) or Aplastic anemia/MDS with PNH clone. Diagnosed cases of aplastic anemia or MDS which evolved to have a PNH clone later during disease course were excluded from the study.

### PNH CASES:

### Classical Hemolytic PNH:

Cases with hypercellular marrow and evidence of biochemical hemolysis (increased LDH and bilirubin) with reticulocytosis and a positive PNH clone were labelled as Classical Hemolytic PNH (CPNH).

### PNH in context of marrow failure:

Cases with BM examination that meet criteria for Aplastic anemia (AA) or Myelodysplastic syndrome (MDS) with variable levels of hemolysis related findings i.e. hemolysis related symptoms, pain, organ dysfunction or variable level of LDH and a positive PNH clone were diagnosed as PNH in context of Marrow failure. There was one patient previously diagnosed as Classical hemolytic PNH which showed evolution to MDS and presented with cytopenias, hypercellular marrow and dysplasia in morphology. This case was also categorized as PNH in context of marrow failure.

### Aplastic anemia with PNH Clone:

Cases diagnosed as aplastic anemia and subclassified as very severe, severe or non-severe based on cytopenias and cellularity of bone marrow as per Camita criteria and found to have a PNH Clone by FLAER were diagnosed as Aplastic anemia with PNH clone.

### MDS with PNH Clone:

Cases diagnosed as MDS based on morphological criteria and positive PNH clone were labelled as MDS with PNH clone.

### FLAER testing:

Approximately 2 ml venous sample was taken in EDTA tubes. For each sample, 5 microliters of FLAER-ALEXA-488/ CD 157-PE/CD 64-ECD/CD 15-PC5/ CD45-PC7 was pipetted out in one tube while 5 microliters of 235a-FITC/CD59-PE was added in the other tube. Neutrophils and monocytes were analyzed for GPI linked CD 157 by adding FLAER combined with CD45, CD15, CD157 and CD64 while RBC population was gated by using CD 235a and co expression of CD 59 was noted. A minimum of 50000 events were analyzed during acquisition. 0.01% was taken as a threshold for PNH positivity. PNH clone was labelled as large (>50%) or small (<30%) based on ASH education program 2021.^10^

### Clinical and Lab correlation:

Clone size was correlated with history of thrombosis, blood counts, hemolytic markers (reticulocyte count, LDH and bilirubin) and final diagnosis i.e. Subtype of PNH, AA/MDS with PNH clone.

### Statistical analyses:

Statistical analyses were performed using SPSS v 25.0. Spearman’s rank correlation was calculated. Multivariate ANOVA analysis was done to determine significance of clinical parameters with size of PNH clone. P-value <0.05 was considered statistically significant.

## RESULTS

Three hundred twenty-four samples were submitted for PNH analysis by FLAER, 96 (29.6 %) found to have PNH clone. Out of these 96 cases, data of 75 (78.12%) cases was available for final analysis. Details of clinical characteristics of patients and clone size is given in Table-I.

**Table-I:** Clinical Characteristics and Clone Size in PNH patients.

Characteristics	Details
Number of patients (n=324)	96 (29.6%) PNH positive cases
Positive PNH cases (n=96)	75(78.1%) evaluable
Age (Mean ± SD)	30.97 ± 10.56
*Gender*	N (%)
Male	60(82.2%)
Female	15(17.3%)
*Complete Blood Count Parameters*	*Median (Range) (IQR)*
Hemoglobin (g/dl)	8.4 (6.8-9.2) (2.4)
Platelet count (x10^9^/l)	111 (35-184) (149)
Absolute Neutrophil Count (x10^9^/l)	2.4 (1.0-3.5) (2.5)
Reticulocyte count (%)	4.1 (2.7-8.7) (6)
LDH (U/l)	698(134-2593) (2459)
Bilirubin (μmol/l)	18 (7.5-34.0) (26.5)
*Diagnosis Subtype*	*N (%)*
**1.PNH:** A.Classical Hemolytic PNH (CPNH)	55(73.3%)
B.PNH in context of marrow failure	16(21.3%)
2.Aplastic anemia with PNH Clone	3(4%)
3.MDS with PNH clone	1(1.3%)
Thrombosis	6 (8%)
*Clone Size*	*N (%)*
***Granulocytes***	
Large	61(81.3%)
Small	14(18.6%)
***Monocytes***	
Large	55(73.3%)
Small	20(26.6%)
RBCs	
Large	28(37.3%)
Small	47(62.6%)

All cases of CPNH had hypercellular marrows for age. Out of 16 cases of PNH in context of marrow failure, 15 had a hypocellular marrow while one had a hypercellular marrow. This patient was a previously known case of CPNH who had developed cytopenias with significant dysplasia over time. Mean age of patients was 30.97 + 10.56 years. 60 (82.2%) patients were male and 15 (17.3%) were female. Granulocyte clone size correlated significantly with monocyte clone size (r= 0.352, p=0.041). Granulocyte clone size was significantly associated with Hb and platelet count (p-values 0.027 and 0.045 respectively). Similarly, there was a significant association found between monocyte clone size and Hb and platelet count (p values 0.013 and 0.017 respectively). However, no significant association of RBC clone size with blood counts could be established. (Table-II).

**Table-II:** Association of Clone size with Blood counts and Markers of Hemolysis.

*Association of Clone size with CBC and Markers of Hemolysis*								
Granulocyte Clone		LDH	BILI	RETIC	Hb	WBC	ANC	PLT
Large (>50%)	Median (Range)	679.5(200-4564)	30.5 (10-110)	4.0(0.3-15)	8.05(3.04-12.9)	4.2(1.6-23.8)	2.7(0.1-20.6)	121.5(5-524)
Small (<30%)	Median (Range)	574.5(349-800)	43.0 (26.6-60)	2.7(0.8-9.0)	9.4(5.4-16.1)	4.0(1.7-12.8)	0.99(0.1-8.74)	18 (11-160)
p-value		0.438	0.83	0.467	0.027	0.641	0.400	0.045
*Monocyte Clone*		*LDH*	*BILI*	*RETIC*	*Hb*	*WBC*	*ANC*	*PLT*
Large (>50%)	Median (Range)	698 (200-3970)	30.5 (10-110)	4.0 (0.3-15.0)	8.2 (4.6-12.9)	4.1 (1.6-23.8)	2.4 (0.1-20.6)	140 (5-367)
Small (<30%)	Median (Range)	375 (317-800)	26.0 (17-60)	3.6 (0.8-9.0)	9.1 (5.4-16.1)	4 (1.7-12.8)	1.2 (0.1-8.7)	22.5 (11-160)
p-value		0.195	0.776	0.538	0.013	0.548	0.558	0.017
*RBC Clone*		*LDH*	*BILI*	*RETIC*	*Hb*	*WBC*	*ANC*	*PLT*
Large (>50%)	Median (Range)	1147 (440-4564)	30.5 (18-42)	4.2 (2.3-9.10)	7.6 (3.4-10.8)	4.8 (2.6-23.8)	2.9 (0.5-20.6)	176 (38-367)
Small (<30%)	Median (Range)	800 (200-8000)	51.5 (10-130)	5.0 (0.5-25)	8.5 (4.6-16.10)	4.16 (1.6-12.85)	2.4 (0.1-9.13)	85 (5-363)
p-value		0.811	0.218	0.393	0.184	0.078	0.233	0.075

Clone size either on WBCs or RBCs showed a significant association with diagnostic subtype of PNH, with majority of cases with Classical Hemolytic PNH having large clone size. (p-value 0.002) However, no association could be elicited between clone size and thrombosis or hemolytic markers, (Table-III).

**Table-III:** Association of clone size with diagnosis and thrombosis.

		Clone size Granulocytes			Clone size Monocytes			Clone size RBCs		
		Large n(%)	Small n(%)	p-value	Large n(%)	Small n(%)	p-value	Large n(%)	Small n(%)	p-value
H/O thrombosis	Yes	6(8)	55 (73.3)	0.276	5(6.6)	50(66.6)	0.489	3(4)	3(4)	0.399
	No	0(0)	14(18.6)		1(1.3)	19(25.3)		25(33.3)	44(58.6)	
PNH sub type	CPNH	53(96.3)	2(3.6)	0.000	46 (83.6)	9(16.4)	0.001	27(49.1)	28(50.9)	
	PNH in context of marrow failure	9 (56.3)	7(46.6)		9(56.3)	7(46.6)		1(6.6)	15(93.8)	0.002

***Note:*** Chi- square test was applied.

## DISCUSSION

The objective of the study was to analyze the impact of clone size on clinical and laboratory presentation of PNH at diagnosis. PNH clones have also been reported to impact the response to immunosuppressive therapy.^11^-^13^ Flow cytometry and FLAER analysis have been used worldwide for the detection of PNH clones. FLAER is a highly sensitive and specific flow cytometry technique which utilizes the binding of florescence labelled proaerolysin to GPI linked proteins on the surface of cell membranes. It can detect PNH clones up to the size of 0.01%.^14^

In the literature, the frequency of PNH clone detected in aplastic anemia and non-aplastic anemia cases has been reported to be 12.02 and 3.36 %, respectively. Introduction of FLAER as a diagnostic modality has increased detection of PNH clones from 4.5 % in the pre-FLAER era to 9.2 %. Some studies also report that aplastic anemia patients have a larger clone size as compared to MDS patients.^15^-^16^ Here, we report 29.6% PNH positivity in samples submitted for FLAER analysis. These included suspected cases of PNH, Aplastic anemia and MDS. Among the evaluable cases, 55 (71.2%) were diagnosed as classical hemolytic PNH(CPNH), 16 (21.3%) as PNH in context of marrow failure, 3 (4.2%) as aplastic anemia with PNH clone and 1 (1.4%) as MDS with PNH clone.

In contrast to our findings, Gupta et al. reported a total of 101 samples including 23 cases of PNH, 46 of aplastic anemia and seven myelodysplastic syndrome cases and 25 normal controls. They reported PNH clone in 23 (50%) of aplastic anemia patients and 3 (42.9%) of MDS patients. While they have reported a median age of 30 (12–55) years in PNH cases and a median age of 27 (5–68) years in aplastic anemia patients, mean age of our patients across all disease groups was 30.97±10.56 years.^4^ Fattizzo et al.^1^ report a median age of 53 years (0–91, IQR 29) and male to female ratio of 1.08. Our study population presented in a relatively younger age group as compared to theirs with a male to female ratio of four. They reported a high prevalence (25%) of PNH clones in aplastic anemia and MDS. Majority of these patients had small (<10%) or very small clone size (<1%). According to them, 176 MDS cases (20.3%) had PNH clone while 327 patients (61.6%) of aplastic anemia displayed a PNH clone. The discordance of PNH clone in aplastic anemia between their study and ours is probably due to the fact that 21.3% of our patients who had a hypocellular marrow with PNH clone were diagnosed as PNH in context of marrow failure instead of aplastic anemia with PNH clone. While they report 20.3% of MDS cases to have a PNH clone, we had only one case of MDS during the study period which developed PNH clone over the course of disease.^1^

At our center, we found that granulocyte clone size correlated significantly with monocyte clone size (r= 0.352, p=0.041), highlighting the fact that both cell lines originate from a common hematopoietic progenitor. We further observed that clone size was significantly associated with PNH sub type with majority of classical hemolytic PNH cases having a large clone size. Granulocyte and monocyte clone size were significantly associated with Hb and platelet count. However, no significant association of RBC clone size with cytopenias could be established. Moreover, we were not able to elicit any significant association between clone size and thrombosis as well. Association of granulocytic clone size with development of thrombosis has been reported in in literature.^17^,^18^

Our findings and those reported by Gupta et al.^4^ in 2010, in this aspect may point towards different disease biology in Southeast Asian population leading to a lower risk of thrombosis as comparted to Western population. Similar to our findings, Gupta reported a significant correlation between PNH clone size and the hemoglobin of patient (r=−0.523; p< 0.05). Similar findings were documented by Pramoonjago et al. and Piedras. However, similar to our study, no significant correlation was found between clone size and hemolytic parameters^19^,^20^ Our findings were contrary to those documented by Fattizzo et al. who reported a significant correlation between clone size and LDH, platelet counts and thrombosis.^1^

Aplastic anemia has typically been associated with PNH clone sizes of <10% at the time of diagnosis.^21^ However, no consensus guidelines are available to label cases with PNH clone >10% as PNH in context of marrow failure or Aplastic anemia with PNH clone. Clinical significance of such a classification in context of Hematopoietic Stem Cell Transplant (HSCT) is yet to be ascertained. 15 (20.6%) of our cases, labelled as PNH in context of marrow failure had cytopenias and hypocellular marrows with increased reticulocyte count with no other evidence of hemolysis. 8 (53.3%) of these cases had a large clone on granulocytes and monocytes and 14 cases (93.3%) had a small clone on RBCs. Three of our cases had cytopenias and hypocellular marrows, fulfilling the Camita criteria with no evidence of hemolysis. They were diagnosed as aplastic anemia with PNH clone. Relative escape of GPI deficient stem cells from the immune attack of cytotoxic T cells might be one of the possible mechanisms underlying evolution of PNH clone in these patients. One patient who was a diagnosed case of MDS on basis of WHO diagnostic criteria showed a PNH clone. This patient had a hypercellular marrow with cytopenias and an increased reticulocyte count with no other markers of hemolysis. Gupta did not clearly define the six patients with >10% PNH clone as PNH in context of marrow failure.^4^

We also compared our results with De Latour et al who analyzed a total of 2489 patients. According to their findings, the majority of patients in the overall study population were female (54.0% [1343/2489]). Mean (standard deviation [SD]) age at PNH start ranged from 36.4 (16.53) to 44.2 (20.70). In contrast to our study, they found a statistically significant difference in mean LDH according to the clone size. (*p*<0.0001). No difference in hemoglobin level at last follow-up was observed in the smaller clone size cohorts, although mean hemoglobin level was lower in patients with clone size >50% (*p*<0.0001). They also reported statistical significance between clone size and Hb, platelet count, ANC and thrombosis.^22^ All these patients are under a regular follow up and a study evaluating the correlation between clone size and response to immunosuppressive therapy with transplant outcome is undergoing in our institute.

### Limitations:

Since the patients were not followed up for longer duration of time, correlation between clone size and evolution of new developing thrombosis or hemolysis could not be ascertained. Moreover, response of immunosuppressive therapy and its correlation with clone size needs to be evaluated through further studies. The only complication that we studied with PNH clone was thrombosis which was found to have no association with clone size. Recommendations about the group of patients requiring close monitoring and their prevention require further studies.

## CONCLUSION

This is probably the first study carried out in Pakistani population reporting the prevalence of PNH clone through a highly sensitive and reliable technique of FLAER analysis and determining its association with biochemical markers of hemolysis, CBC and thrombosis. The study highlights the role of advanced diagnostic modalities which may tailor patient management and affect the outcome and also help us in understanding the disease pathophysiology.

### Recommendations:

Based on our findings, FLAER should be used as a recommended method for screening in suspected cases of PNH. Patients with cytopenias and with hemolysis/ reticulocytosis must be screened for PNH clones. Similarly, all cases of aplastic anemia and MDS should be observed for development of PNH clone over the course of the disease. PNH screening should be considered in all young patients presenting with thrombosis.
